# Modelling Future Coronary Heart Disease Mortality to 2030 in the British Isles

**DOI:** 10.1371/journal.pone.0138044

**Published:** 2015-09-30

**Authors:** John Hughes, Zubair Kabir, Kathleen Bennett, Joel W. Hotchkiss, Frank Kee, Alastair H. Leyland, Carolyn Davies, Piotr Bandosz, Maria Guzman-Castillo, Martin O’Flaherty, Simon Capewell, Julia Critchley

**Affiliations:** 1 UKCRC Centre of Excellence for Public Health, Queen’s University, Belfast, United Kingdom; 2 Department of Epidemiology &Public Health University College Cork, Cork, Ireland; 3 Department of Pharmacology and Therapeutics, Trinity Centre for Health Sciences, St James’s Hospital, Dublin, Ireland; 4 School of Veterinary Medicine, University of Glasgow, Glasgow, United Kingdom; 5 MRC/CSO Social and Public Health Sciences Unit, University of Glasgow, Glasgow, United Kingdom; 6 Department of Public Health & Policy, Institute of Psychology, Health & Society, University of Liverpool, Liverpool, United Kingdom; 7 Population Health Research Institute, St Georges University of London, London, United Kingdom; University of Louisville, UNITED STATES

## Abstract

**Objective:**

Despite rapid declines over the last two decades, coronary heart disease (CHD) mortality rates in the British Isles are still amongst the highest in Europe. This study uses a modelling approach to compare the potential impact of future risk factor scenarios relating to smoking and physical activity levels, dietary salt and saturated fat intakes on future CHD mortality in three countries: Northern Ireland (NI), Republic of Ireland (RoI) and Scotland.

**Methods:**

CHD mortality models previously developed and validated in each country were extended to predict potential reductions in CHD mortality from 2010 (baseline year) to 2030. Risk factor trends data from recent surveys at baseline were used to model alternative future risk factor scenarios: Absolute decreases in (i) smoking prevalence and (ii) physical inactivity rates of up to 15% by 2030; relative decreases in (iii) dietary salt intake of up to 30% by 2030 and (iv) dietary saturated fat of up to 6% by 2030. Probabilistic sensitivity analyses were then conducted.

**Results:**

Projected populations in 2030 were 1.3, 3.4 and 3.9 million in NI, RoI and Scotland respectively (adults aged 25–84). In 2030: assuming recent declining mortality trends continue: 15% absolute reductions in smoking could decrease CHD deaths by 5.8–7.2%. 15% absolute reductions in physical inactivity levels could decrease CHD deaths by 3.1–3.6%. Relative reductions in salt intake of 30% could decrease CHD deaths by 5.2–5.6% and a 6% reduction in saturated fat intake might decrease CHD deaths by some 7.8–9.0%. These projections remained stable under a wide range of sensitivity analyses.

**Conclusions:**

Feasible reductions in four cardiovascular risk factors (already achieved elsewhere) could substantially reduce future coronary deaths. More aggressive polices are therefore needed in the British Isles to control tobacco, promote healthy food and increase physical activity.

## Introduction

Coronary Heart Disease (CHD) mortality rates have more than halved in Northern Ireland, [1] Republic of Ireland [2] and Scotland [3] as in other European countries over the last four decades. However, CHD remains a leading cause of death and disability and countries in the British Isles report unacceptable levels of premature CHD mortality compared to other Northern and Western European countries. [4]

However, other countries have been more successful at reducing smoking prevalence to less than 15%, (Australia) [5] decreasing dietary salt intake (by 3g-5g) (Japan) [6] and saturated fats (Finland), [7] and increasing physical activity (Poland). [8]

The objective of this study was therefore to evaluate the impact of a number of cardiovascular risk factor changes on future levels of CHD mortality to 2030 in three countries in the British Isles which have separate policy making frameworks.

The IMPACT model quantifies observed decreases in CHD mortality which can be attributed to (i) risk factor changes in the population and (ii) advances in evidence based medical and surgical treatments. IMPACT models for Northern Ireland (1987–2007) and the Republic of Ireland (1985–2006) were validated to assess their performance in predicting observed mortality in 2010.

In this paper we extended established IMPACT models developed in Northern Ireland, Republic of Ireland and Scotland to examine the effect of a number of improved population level risk factor scenarios on projected CHD Mortality in 2030.

## Methods

### The IMPACT model

Original IMPACT models for Northern Ireland (NI) (1987–2007) and the Republic of Ireland (RoI) (1985–2006) were validated using the most recent year of the original model analysis, as a base year (i.e. 2006 or 2007), to predict age and gender stratified CHD deaths in 2010. The projected number of deaths was compared to the observed or actual number of CHD deaths in 2010 (ICD-10 codes I20-I25). “Table A in S1 Appendix”. Updated IMPACT models for each country were extended from a baseline year of 2010 to predict the number of deaths in the year 2030. We then estimated the impact on future CHD mortality of reductions in smoking prevalence, reductions in prevalence of physical inactivity, reductions in systolic blood pressure due to a fall in salt intake in the population and reduction in total cholesterol level due to replacing diet energy from saturated fats by polyunsaturated fats or mono unsaturated fats.

### Data sources

Data were sourced for each country stratified by gender and by 10-year age group for persons aged 25–84 years “Tables B and C in S1 Appendix”). For the baseline year of the prediction model (2010) representative national surveys were used to source risk factor data for each country. Mortality data for 2010, population estimates for 2010 and population projections for 2030 were obtained from official statistics agencies, the Northern Ireland Statistics & Research Agency, Central Statistics Office and National Records of Scotland, Scottish Government for NI, ROI and Scotland respectively. To estimate population change in CHD mortality, regression beta coefficients to quantify the impact of smoking and physical inactivity were sourced from larger cohort studies. [9,10] Regression coefficients from meta-analyses of cohort studies were used to quantify the mortality effects of population reductions in salt intake [11] and on the effect of replacing dietary saturated fat intake by either polyunsaturated fats or monounsaturated fats. [12]

### Estimating future CHD mortality to 2030

Future CHD mortality in 2030 was estimated using two approaches. Firstly, ‘lower mortality’ values for 2030 were determined by fitting age and gender specific negative exponential decay models where future mortality decays at a rate directly proportional to historical CHD mortality. Our models used observed CHD mortality rates from 1995–2010 to determine the rate of decay. An iterative nonlinear least squares model: y = a*exp (-b*year) was fitted to predict future CHD mortality from 2010 through 2030 where a is the CHD mortality rate and b is the constant decay rate “Figure A in S1 Appendix”. Secondly, ‘no mortality change’ 2030 CHD mortality values were estimated using an indirect standardisation approach by multiplying individual country age and gender specific mortality rates for 2010 by the projected population for each 10-year age group in the year 2030.

### Estimating future CHD mortality with enhanced risk factor reductions

#### (a) Reductions in smoking and physical inactivity

Using regression coefficients from the literature a population attributable risk fraction (PARF) approach was used to determine the number of deaths prevented or postponed (DPPs) in 2030 resulting from alternative improved future smoking and physical activity levels. [13,14] The PARF was calculated conventionally for 2010 and 2030 as (P x (RR-1)) / (1+P x (RR-1)) where P is the prevalence of the risk factor and RR is the relative risk for CHD mortality associated with the individual risk factor “Example A in S1 Appendix” The number of deaths prevented was calculated as the number of deaths in 2030 multiplied by the decrease in PARF between 2010 and 2030.

#### (b) Reductions in cholesterol and systolic blood pressure

**Fig 1:**
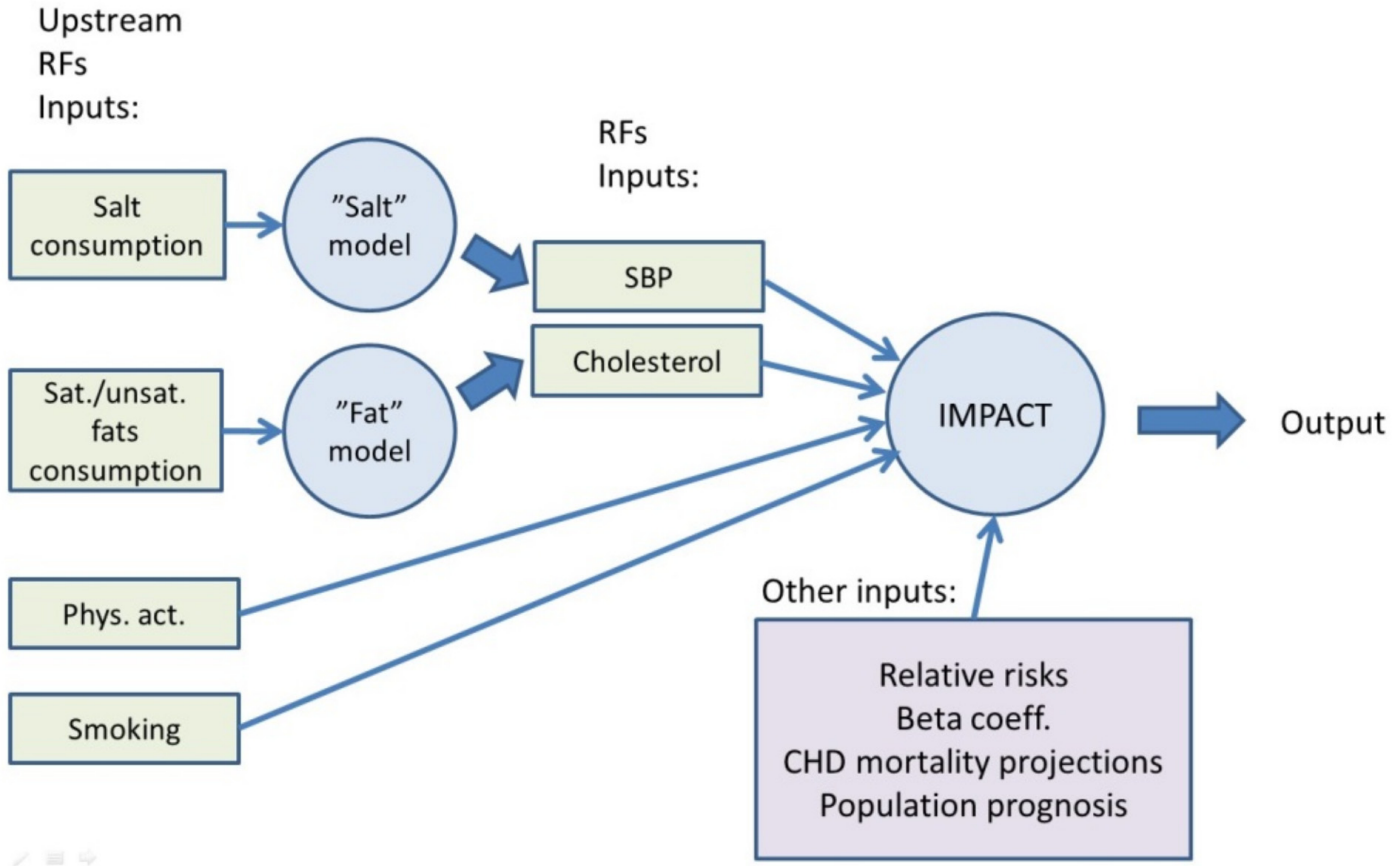
Updated IMPACT CHD Model.

The effect of saturated fat intake on serum cholesterol levels were estimated using the Clarke equations [12] to translate a change in saturated fat intake into a change in total cholesterol levels, assuming iso-caloric replacement with polyunsaturated and mono-saturated fats (assuming that each 1% absolute reduction in energy from saturated fat was replaced by 0.1% energy from mono- and 0.9 energy from poly-unsaturated fats). The differential effect of salt reduction in the diet to 2030 on systolic blood pressure in hypertensive and normotensive persons was taken from a large meta-analysis. [11] Conventional IMPACT methodology was then used to translate the change in systolic blood pressure levels into mortality reductions “Table D in S1 Appendix”. The updated IMPACT model is shown in Fig 1.

For all risk factor scenarios under consideration, separate calculations for projected CHD deaths in 2030 were undertaken based on two contrasting mortality scenarios, a) a lower mortality assumption and b) conservatively assuming ‘no mortality change’ (not shown).

### Cumulative benefit of multiple risk factor changes

As CHD deaths are usually caused by multiple risk factors acting together, mortality benefits from risk factor reductions occurring simultaneously were estimated using the cumulative risk-reduction approach. [13] This assumes a less than additive effect of multiple risk factor changes occurring simultaneously.

### Estimating reductions in CHD deaths in 2030

Both ideal (scenario 1) and modest (scenario 2) risk factor improvements were modelled. The ideal scenarios, although more challenging, are feasible as they have been achieved in other countries. [7–10]

### Scenario 1 –‘Ideal’ improvements in risk factors by 2030

- absolute smoking prevalence to reduce by 15%,
- absolute physical inactivity rates to reduce by 15%,
- relative decreases in dietary salt intake of up to 30% and
- decreases in dietary saturated fat intake of up to 6% (absolute decreases in percentage energy intake from saturated fats, replaced by unsaturated fats).

### Scenario 2- ‘Modest’ improvements in risk factors by 2030

- absolute smoking prevalence to reduce by 5%
- absolute physical inactivity rates to reduce by 5%
- relative decreases in dietary salt intake of up to 10% and
- decreases in dietary saturated fat of up to 2% (absolute decreases in percentage energy intake from saturated fats, replaced by unsaturated fats).

### Extrapolation of estimates to British Isles Population

Estimated reductions in future CHD deaths for all countries in the British Isles to 2030 were simply calculated for the combined reduction of the four risk factors under consideration. Scottish projections were extrapolated to estimate deaths prevented for the populations of England and Wales.

### Sensitivity analyses

Uncertainty in modelling estimates was quantified by running an iterative Monte Carlo simulation using R software (R Core Team (2014). R: A language and environment for statistical computing. R Foundation for Statistical Computing, Vienna, Austria. URL http://www.R-project.org/). The model was repeated 10,000 times, using random numbers drawn from specific distributions as model input values. We calculated the uncertainty intervals based on 10,000 draws taking the 95% uncertainty intervals as the 2.5th and 97.5th percentiles. Input variables taken from external sources (e.g. beta coefficients and relative risk reductions) were randomly drawn from specified distributions. The distributions used for the main input parameters are detailed in “Table L in S1 Appendix”.

### Ethics Statement

Our study is a secondary data analyses of routinely available mortality, population and Health Survey data. It thus only uses anonymised data, and additional ethical approval was therefore not required.

## Results

A validation exercise using risk factor and treatment values for the final year of original IMPACT models for NI & RoI to predict CHD deaths in 2010 showed high levels of agreement for NI (91%) & RoI (106%) between the predicted and actual number of CHD deaths “Table B in S1 Appendix”.

**Fig 2:**
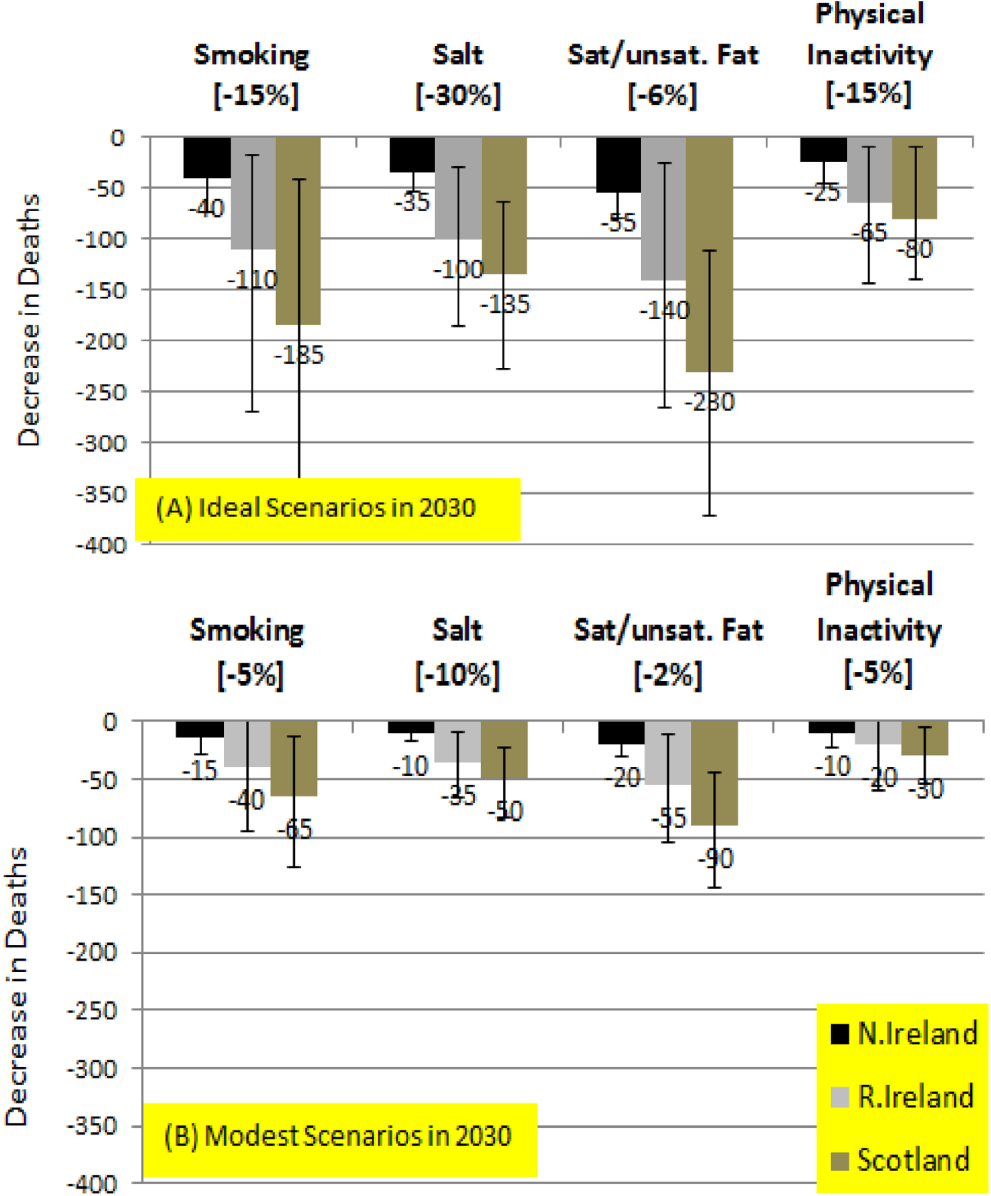
Predicted Decreases in Deaths in 2030 based on ‘Lower CHD mortality in 2030’–IDEAL Scenarios (the error bars show the extreme minimum and maximum values in the sensitivity analysis.

Table 1 details baseline (2010) and future (2030) risk factor weighted average values (25–84 years) used in the models for each country. Table 2 reports modelled estimates of potentially preventable CHD deaths (expressed as a percentage of total expected deaths in 2030) pertaining to each future risk factor scenario. For a ‘lower CHD mortality in 2030’ assumption, estimates of the number of CHD deaths that could potentially result from achievable ‘modest’ (scenario 1) and more optimistic ‘ideal’ (scenario 2) future risk factor scenarios are shown in Fig 2. Separate modelling estimates based on assuming a no CHD mortality change to 2030 were also quantified “in Table J and Figure B in S1 Appendix”.

**Table 1:** Weighted average of risk factor levels used for 2010 baseline and for 2030 projections for adults aged 25–84 years.

	NI			ROI			Scotland		
	2010	2030*	2030**	2010	2030*	2030**	2010	2030*	2030**
**Smoking (%)**	24.1	17.7	8.7	28.0	20.4	10.7	25.4	19.5	9.8
Physical Inactivity(%)***	42.3	39.4	29.4	41.2	37.8	27.8	62.9	59.7	49.7
**Total Cholesterol (mmol/l)**	5.2	5.0	4.7	5.2	5.0	4.7	5.3	5.2	4.9
**SBP (mmHG)**	127.8	129.0	127.7	127.0	129.0	127.7	129.1	129.3	128.0

*Scenario 1 modest scenarios

** Scenario 2 Ideal Scenarios

*** At least 150 minutes moderate activity per week or 60 minutes vigorous activity per week (or a combination of the two)

**Table 2:** Predicted decreases in deaths expressed as a percentage of total expected CHD deaths in 2030 –Ideal & Modest Future Risk Factor Scenarios.

	Predicted percentage* DPPAssumed lower mortality in 2030		
	NI	RoI	Sco
**Ideal Scenarios**			
**Smoking -15%**	5.8 (1.5–11.0)	6.2 (-1.0–15.2)	7.2 (1.6–14.2)
**Salt -30%**	5.3 (3.1–8.1)	5.6 (1.6–10.3)	5.2 (2.4–8.8)
**Sat/ Unsat fats -6%**	8.6 (5.2–12.6)	7.8 (1.4–15.0)	9 (4.4–15.5)
**Phy Inactivity -15%**	3.6 (0.3–6.7)	3.6 (-0.5–8.0)	3.1 (0.3–5.5)
**Modest Scenarios**			
**Smoking -5%**	2.5 (0.6–4.7)	2.3 (-0.3–5.5)	2.6 (0.6–5.0)
**Salt -10%**	1.9 (1.1–3.0)	2.0 (0.5–3.8)	1.9 (0.9–3.3)
**Sat/ Unsat fats -2%**	3.3 (2.0–4.9)	3.0 (0.5–5.7)	3.5 (1.7–5.6)
**Phy Inactivity -5%**	1.2 (-0.5–3.3)	1.3 (-0.5–3.3)	1.1 (0.1–2.1)
**Expected Deaths**	646 (439–860)	1773 (682–2936)	2562 (1324–3916)

*Expressed as a percentage of expected deaths in 2030

### Scenario 1 –Ideal improvements in risk factors by 2030

Based on the lower mortality assumption, a total of 150 (23.3%), 410 (23.2%) & 625(24.4%) deaths could potentially be avoided in NI, RoI and Scotland respectively if ambitious risk factor improvements across the four risk factor scenarios were achieved (See Fig 1). In relative terms, there was greater potential of risk factor improvements to reduce deaths in women (between 25.1% and 25.7%) compared to men (between 22.3% and 23.8%) “Table J in S1 Appendix”. Lowering saturated fat level intake by 6% in absolute terms would result in 55 (8.6%), 140 (7.8%) & 230 (9.0%) fewer CHD deaths in NI, RoI and Scotland respectively (the percentages here are based on overall deaths potentially avoided in each country, Table 2). A substantial but feasible reduction in smoking prevalence of 15% would result in 40 (5.8%) 110 (6.2%) & 185 (7.2%) fewer deaths in NI, RoI & Scotland. There were marked gender differences regarding reduction in deaths due to smoking with greater potential relative reductions in women (6.9%-8%) compared to men (5.4%-6.7%). Reducing salt levels in relative terms by 30% would reduce CHD deaths by 5.2–5.6% (35, 100 & 135 fewer deaths in NI, RoI & Scotland). A slightly higher proportion of deaths could be prevented from reduced salt intake in women (5.7%-5.9%) compared to men (4.9%-5.4%). An absolute improvement in physical activity levels in each country of 15% could potentially prevent 3.1–3.6% of CHD deaths in 2030 (25, 65 & 80 fewer deaths in NI, RoI & Scotland), with no gender differences.

### Scenario 2—Modest improvements in risk factors by 2030

Based on a lower mortality assumption, modest changes across the four risk factor scenarios could result in a total of 60, 150 and 230 fewer deaths in 2030 in NI, RoI and Scotland respectively. The biggest gains would come from a 2% absolute reduction in saturated fat intake levels in decreases in deaths of 3.0–3.5% (approximately 20, 55 & 90 fewer deaths in NI, RoI and Scotland respectively). A 5% absolute decline in smoking by 2030 would translate into 15, 40 and 65 fewer CHD deaths (in percentage terms 2.3–2.6%). As regards salt intake, relative decreases of 10% might decrease CHD deaths by 1.9–2.0% (approximately 10, 35 and 50 deaths in NI, RoI and Scotland respectively). Approximately 10, 20 and 30 fewer deaths in NI, ROI and Scotland respectively (approximate decrease of 1.1–1.3%) could be attributed to a 5% absolute decrease in physical inactivity levels. For all countries, there were more marked relative decreases in deaths in women overall and in particular declines in mortality attributed to reductions in smoking levels and salt intake (Table I in S1 Appendix).

Modelling results based on ‘no mortality change’ scenarios resulted in marginally higher estimates of DPPs when expressed as a percentage of total expected deaths (Table J in S1 Appendix.

### Extrapolation of models estimates to British Isles population projections for 2030

Based on the lower mortality assumption, the Scottish population of 3.9 million (25–84 years) translated into 230 or 625 total CHD deaths prevented resulting from the ‘Modest’ and ‘Ideal’ future risk factor scenarios respectively. Simple extrapolation of deaths potentially prevented from these Scottish estimates to the populations of England and Wales (25–84 years) of 42.4 million suggests that 2480 & 6745 fewer CHD deaths could be expected in the British Isles in 2030 (Table K in S1 Appendix).

## Discussion

Our modelling estimates using data from NI, RoI and Scotland suggest that reducing four selected risk factors could substantially decrease CHD mortality by 2030, by somewhere between 9% (‘modest’ scenarios) and 24% (‘ideal’ scenarios).

In the year 2030 over 6,800 CHD deaths could be avoided in the British Isles by optimal improvements in average population dietary salt and saturated fat intake, reductions in unacceptable high levels of smoking prevalence and increases in physical activity levels. Alternatively, if modest and very feasible improvements in the same risk factors were achieved as in other countries, approximately 2500 CHD deaths could be avoided in the British Isles. There is greater potential to reduce blood pressure levels in older women. Our results also reflect the vast potential to reduce heart disease deaths in both men and in particular women. (aged over 65) resulting from increased post-menopausal risks of CHD.

Previous studies using IMPACT methodology have consistently shown a similar contribution to decreasing CHD mortality trends attributed to reduction in population risk factor levels compared to treatments. [1,2,14] Decreases in blood pressure, total cholesterol and smoking explained more than 60% of the decrease in CHD mortality in NI (1987–2007) [1] in ROI (1985–2006) [2] and contributed to 50% of the CHD mortality decline in an earlier study carried out in Scotland (1975–1984). [3]

Despite marked smoking declines in NI, ROI & Scotland over the last number of decades and repeated evidence of reductions in CHD mortality due to declines in smoking prevalence, current smoking levels in the ‘celtic fringe’ countries remain unacceptably high at approximately 25%. [15]

A large proportion of the blood pressure and cholesterol improvements in the UK over the last 20–30 years can be attributed to changes in diet. [16,17] A recent UK wide ‘family food survey’ (2007–2009) found higher levels of saturated fat & salt intake and lower fruit and vegetable consumption in both NI and Scotland compared to England & Wales. [18] ROI has previously reported better uptake of at least one daily portion of fruit and vegetable intake per day compared to NI. [15]

Although physical inactivity data from NI, RoI and Scotland were derived from the International Physical Activity (IPAQ) questionnaire, [19] markedly higher levels of physical inactivity were reported in Scotland compared to NI & RoI. Higher inactivity in Scotland can be explained by differing interpretations of the IPAQ questionnaire being employed as the Scottish activity figures used are based on previous UK recommendations of performing at least moderate activity lasting 30 minutes per day ≥ 5 days per week whereas NI & RoI data are aligned with current UK recommendations of meeting at least 150 minutes of moderate intensity activities per week. A recent study from the Republic of Ireland reported improving physical activity levels in the Republic of Ireland between 1985 and 2006. [2] As devolved administrations of the United Kingdom (UK), NI and Scotland have responsibility for much of public health policy, although UK wide policy and law still dictate taxation, advertising and consumer protection issues such as health warnings on tobacco products. A renewed commitment to primary prevention and risk factor reduction is central to overarching public health policies and CHD focused strategies in each country. [20–22] Similar population based prevention approaches for smoking have been adopted in all three countries with continued taxation on tobacco products and implementation of smoking bans in enclosed public places (March 2004 in RoI, March 2006 in Scotland and April 2007 in NI). However, there is substantial room for improvement as recent survey data suggest that smoking rates are still much higher in British Isles countries compared to other Northern European countries. It is anticipated that NI & Scotland will eventually follow proposals in ROI to introduce standardised plain packaging of cigarettes. This ground breaking initiative had an immediate positive impact in Australia. [23]

Over the last few decades the UK has had some success in reducing average salt and saturated levels by setting voluntary targets and by improving nutritional labelling indicating the level of saturated fats, salt and sugars on pre-packaged foods. However, average salt intakes in the UK and Ireland are substantially higher than the recommended intake of 6 grams per day. [24] Bigger decreases in CHD mortality would be achieved by mandatory reformulation of processed foods and by introducing subsidies for growing healthier foods. [25] As well as making a huge difference to population health, the implementation of population based mandatory standards for lower levels of salt and saturated fats in processed foods is cost effective. [26] The potential of introducing effective food policies is highlighted by recent studies which quantified the number of CVD deaths that could be avoided in NI and Scotland and in ROI by population level changes in diet. [27]

The overriding goal of current physical activity strategies in NI, RoI and in Scotland remains to increase active travel. Recent UK wide co-operation to set targets of further incorporating physical activity into daily life and to standardize physical activity definitions is encouraging. However, more radical approaches to promote active travel such as financial incentives (selective taxes or subsidies) need to be explored for a sustained population shift from sedentary living to more cycling and walking to be realized. [28]

### Strengths and Limitations

Our study has several strengths including incorporating high quality recent risk factor trend data from high quality national surveys and we cumulative adjusted for multiple risk factor changing simultaneously. As ever with modelling studies there are some limitations. Projections of mortality to 2030 are likely to be speculative and uncertain. However, different approaches to projecting mortality was considered and probabilistic sensitivity analysis performed to ensure robustness of findings. Direct physical activity comparisons between countries are difficult due to self reported physical activity data being prone to bias. [29] However, improved UK-wide physical activity guidelines were published in 2011. Our projections model only estimate future CHD deaths although concurrent reductions in future non fatal CHD events would also be achieved based on the future risk factors scenarios modelled.

As part of our ongoing work we will specifically model potential reductions in CHD mortality resulting from reductions in obesity, or diabetes when there is sufficient evidence of effective population level interventions to reverse recent substantial increases in these important risk factors. Although, population level interventions to reduce obesity and diabetes by 2030 were not included in the current study, the rising levels of these risk factors in our population were taken into account as part of the baseline risk, which was used to project future absolute levels of CHD mortality.

We did not model the effect of improvements in therapies which could also occur and might reduce mortality further. Therefore, the model assumes no change in therapies uptake or effectiveness over time. We did not distinguish between risk factor reduction in ‘healthy subjects’ and persons with recognised heart disease, but at a population level this will have little impact on our estimates.

The current mortality projection method did not take into account competing risks for mortality. However, the model probably underestimates potential gains, as risk factors changes such as smoking would reduce the incidence of related cancers and stroke.

It was assumed that modelled estimates of one individual country in the UK could be extrapolated to the overall UK population and this poses the risk of accentuating any bias in modelling estimates. However, all countries in the British Isles have similar age profiles and cardiovascular risk factors are comparable in all countries. Validation and sensitivity analyses carried out mitigate concerns with comparability with data across all British Isles countries.

A notable strength is the model validation highlighting the high predictive ability of original models (>90%) in projecting mortality to 2010. As CHD mortality rates can change abruptly over a short period of time, two contrasting future mortality scenarios were modelled. However, marked cardiovascular mortality decreases can occur rapidly following risk factor changes in the population and our modelled estimates should not be taken as precise projections but as useful indicators of future CHD mortality which might be expected based on different future risk factor scenarios. [30]

### Conclusions

CHD mortality improvements have been a major success story over recent decades. Yet without further policy action, the future burden and cost of treating CHD could increase exponentially. And CHD is largely preventable. However, radical policies will be needed to further improve diet and reduce smoking in order to minimise the future burden of CHD.

## Supporting Information

S1 Appendix Table A. Validation of IMPACT models, 2010. Fig A. NI CHD Mortality Projections to 2030 for males aged 55–64 using a ‘lower mortality assumption’. Table B. Data sources used in projecting CHD mortality—definitions and data sources for NI, RoI & Scotland. Table C Gender Specific risk factor levels in NI, RoI and Scotland in 2010 by Agegroup. Example A. Estimation of DPPs from risk factor change using PARF method. Table D Beta coefficients for blood pressure change in population. Table E. Beta coefficients for total cholesterol change in population. Table F. Relative risk of mortality from Ischaemic Heart Disease for current smokers relative to non-smokers. Table G. Relative risk of Ischaemic Heart Disease from physical (in)activity levels from WHO GBD Study. Table H Predicted decreases in deaths expressed as a percentage of total expected CHD deaths in 2030 –Ideal & Modest Future Risk factor Scenarios. Table I. Predicted decreases in deaths by gender expressed as a percentage of total expected CHD deaths in 2030 –Ideal & Modest Future Risk factor Scenarios (Assumed lower mortality). Table J. Predicted decreases in deaths by gender expressed as a percentage of total expected CHD deaths in 2030 –Ideal & Modest Future Risk factor Scenarios (No mortality change). Fig B. Predicted decreases in Deaths in 2030 based on ‘No mortality change’ between 2010 and 2030 for (A) IDEAL scenarios & (B) MODEST Scenarios. Table K. Extrapolation of modelled estimates to the British Isles population for 2030. Table L. Distributions used for main input parameters in the model.
(DOCX)
