# Supplementary material for: Modelling Future Coronary Heart Disease Mortality to 2030 in the British Isles
Source: PLoS One. 2015 Sep 30;10(9):e0138044. doi: 10.1371/journal.pone.0138044 (PMC4589484; doi:10.1371/journal.pone.0138044)
Supplement: S1 Appendix — Table A. Validation of IMPACT models, 2010. Fig A. NI CHD Mortality Projections to 2030 for males aged 55–64 using a ‘lower mortality assumption’. Table B. Data sources used in projecting CHD mortality—definitions and data sources for NI, RoI & Scotland. Table C Gender Specific risk factor levels in NI, RoI and Scotland in 2010 by Agegroup. Example A. Estimation of DPPs from risk factor change using PARF method. Table D Beta coefficients for blood pressure change in population. Table E. Beta coefficients for total cholesterol change in population. Table F. Relative risk of mortality from Ischaemic Heart Disease for current smokers relative to non-smokers. Table G. Relative risk of Ischaemic Heart Disease from physical (in)activity levels from WHO GBD Study. Table H Predicted decreases in deaths expressed as a percentage of total expected CHD deaths in 2030 –Ideal & Modest Future Risk factor Scenarios. Table I. Predicted decreases in deaths by gender expressed as a percentage of total expected CHD deaths in 2030 –Ideal & Modest Future Risk factor Scenarios (Assumed lower mortality). Table J. Predicted decreases in deaths by gender expressed as a percentage of total expected CHD deaths in 2030 –Ideal & Modest Future Risk factor Scenarios (No mortality change). Fig B. Predicted decreases in Deaths in 2030 based on ‘No mortality change’ between 2010 and 2030 for (A) IDEAL scenarios & (B) MODEST Scenarios. Table K. Extrapolation of modelled estimates to the British Isles population for 2030. Table L. Distributions used for main input parameters in the model. (DOCX) [file pone.0138044.s001.docx]

**S. Supplementary Information**

# Table of Contents

**Table A:** Validation of IMPACT models, 2010....................................................................................Page 3

**Figure A:** NI CHD Mortality Projections to 2030 for males aged 55-64 using a ‘lower mortality

assumption’.............................................................................................................................................Page 4

**Table B:** Data sources used in projecting CHD mortality - definitions and data sources for

NI, RoI & Scotland................................................................................................................................Page 5

**Table C:** Gender Specific risk factor levels in NI, RoI and Scotland in 2010 by agegroup...............Page 7

**Table D:** Beta coefficients for blood pressure change in population....................................................Page 10

**Table E:** Beta coefficients for total cholesterol change in population.................................................Page 10

**Table F:** Relative risk of mortality from Ischaemic Heart Disease for current smokers relative to non-smokers...........................................................................................................................Page 11

**Table G:**  Relative risk of Ischaemic Heart Disease from physical (in)activity levels from WHO GBD Study..................................................................................................................................Page 11

**Table H:** Predicted decreases in deaths expressed as a percentage of **total** expected CHD deaths in 2030 – Ideal & Modest Future Risk factor Scenarios............................................................Page 12

**Table I:** Predicted decreases in deaths by **gender** expressed as a percentage of **total** expected CHD deaths in 2030 – Ideal & Modest Future Risk factor Scenarios..(Assumed lower mortality)...............Page 13

**Table J:** Predicted decreases in deaths by **gender** expressed as a percentage of **total** expected CHD deaths in 2030 – Ideal & Modest Future Risk factor Scenarios (No mortality change).......................Page 14

**Figure B:** Predicted decreases in Deaths in 2030 based on ‘No mortality change’ between 2010 and 2030 for (A) IDEAL scenarios & (B) MODEST Scenarios...................................Page 15

**Table K:** Extrapolation of modelled estimates to the British Isles population for 2030.....................Page 16

**Table L:** Distributions used for main input parameters in the model.................................................Page 17

S1. IMPACT MORTALITY PROJECTIONS 2030 MODELS: INTRODUCTION and DETAILED METHODOLOGY

The tables included in this supplementary appendix document provide details about the methods that were used in creating IMPACT mortality projections models for Northern Ireland (NI), Republic of Ireland (RoI) and Scotland. These models estimate the future impact on Coronary Heart Disease (CHD) mortality by 2030 of changes in smoking prevalence, changes in prevalence of physical inactivity, changes in systolic blood pressure due to reduction of salt intake in the population and changes in total cholesterol level due to replacing diet energy from saturated fats by polyunsaturated fats or mono unsaturated fats.

Original IMPACT mortality models quantifying observed decreases in CHD mortality which can be attributed to (i) risk factor changes in the population and (ii) advances in evidence based medical and surgical treatments were developed for NI (1987-2007)^1^, ROI (originally 1985-2000^2^ & then subsequently 1985-2006^3^) & Scotland (1975-1999^4^). Original cell-based ‘historical’ IMPACT mortality model, developed in Microsoft Excel, have been described in detail online and elsewhere.^1, 2,3^

**S2. Validation of IMPACT models**

IMPACT models in NI (1987 -2007) & RoI (1985-2006) were validated using the most recent year of the original model analysis, as a base year, to predict age and gender stratified CHD deaths in 2010. The predicted number of CHD deaths was then compared to the observed number of CHD deaths in 2010 to ascertain how well the models estimated the number of deaths in 2010. The overall results (for person aged 25-84) of this validation exercise are displayed in Table A.

| Country | **Base year of original model** | **End year of original model** | **Model Estimate of 2010 deaths** | **Observed estimate of 2010 deaths** | **% agreement** |
| --- | --- | --- | --- | --- | --- |
| NI | 1987 | 2007 | 1,360 | 1,492 | 91% |
| RoI | 1985 | 2006 | 3,150 | 2,966 | 106% |

**Table A: Validation of IMPACT models, 2010***

*****As there was substantial development to the IMPACT model since the end year of the Original Scotland IMPACT model (1999) it was not meaningful to use the original Scotland (1975-1999) IMPACT model for validation purposes.

**S3. Projected Changes in CHD mortality rates by 2030**

Estimates of 2030 CHD Mortality were calculated using two distinct approaches using (1) a lower mortality assumption and (2) a ‘no mortality change’ assumption.

**S3A. Lower Mortality Assumption**

A number of models were assessed to explain past trends in age and gender stratified CHD mortality (ICD-9 codes 410-414 and ICD-10 codes I20-I25). A negative exponential decay model Future CHD mortality in 2030 was estimated using two approaches. Firstly, ‘lower mortality’ values for 2030 were determined by fitting age and gender specific negative exponential decay models where future mortality decays at a rate directly proportional to historical CHD mortality . Our models used observed CHD mortality rates from 1995-2010 to determine the rate of decay b. An iterative nonlinear least squares model: y=a*exp(-b*year) was fitted to predict future CHD mortality from 2010 through 2030 where a is the CHD mortality rate and b is the constant decay rate. S1 Figure illustrates the past and future CHD mortality in NI for males aged 55-64.

Y= 610.30*exp (-.07*year)

**Figure A. NI CHD Mortality projections to 2030 for males aged 55-64 using a ‘lower mortality’ assumption.**

**S3B.** **No Mortality Change Assumption**

Age-standardization was undertaken using the indirect method based on 2010 mortality rates for each individual country. Gender and age- specific CHD mortality rates from 2010 were applied to corresponding projected population estimates for 2030 in each country. Summing over all age strata then yielded the predicted numbers of deaths from CHD in 2030.

**S4. Data sources**

|  | **Northern Ireland** | | **Republic of Ireland** | | **Scotland** | |
| --- | --- | --- | --- | --- | --- | --- |
|  | ***Definition*** | ***Source*** | ***Definition*** | ***Source*** | ***Definition*** | ***Sourceee*** |
| **Systolic blood pressure** | Measured SBP | NANS 2008-10  &TILDA 2010 | Measured SBP | NANS  08-10  &TILDA 2010 | Measured | SHeS |
| **Mean Total Cholesterol** | Measured Chol | SLAN 2007 | Measured Chol | SLAN 2007 | Measured | SHeS |
| **Hypertension** | SBP ≥140mmHg or  DBP ≥ 90 mmHg or  on treatment for hypertension | HSFe 2010 | NANS- WHO criteria  (SBP > 140mmHg )  TILDA- SBP ≥140mmHg or  DBP ≥ 90 mmHg | NANS 2008-10  & TILDA (50+ years of age) 2008 | SBP ≥140mmHg or  DBP ≥ 90 mmHg, irrespective of treatment | SHeS |
| **Smoking** | Current smoker | HSNI 2010/11 | Current smoker | SLAN 2007/ TILDA 2008 | Current smoker | SHeS |
| **Physical inactivity*** | Not meeting recommendations of ≥ 5 occassions/week of at least moderate activity ( for at least 30 minutes per day) | HSNI 2010/11 | At least 150 mins moderate activity per week or 60 mins vigorous activity per week ( or a combination of the two) | SLAN 2007 | At least 150 mins moderate activity per week or 60 mins vigorous activity per week ( or a combination of the two) | SHeS |
| **Mean salt intake** ( g/ per day) | (g/day) – includes both household & purchases eaten outside home | FFS 2009-11  (3 year average) | Spot Urine analysis- mean g/day (SD) | SLAN 2007 | 24 hour urine collection SHeS | SHeS |
| **CHD Deaths 2010** | ICD10 I20-I25 | NISRA | ICD10  I20-I25 | CSO | ICD10 I20-I25 | NRS |
| **Population estimates 2010** | Mid-year population estimates 2010  ( used revised figures following 2011 census updates) | NISRA | Estimates based on residence concept (projected on 2006 census) | CSO | Mid-year population estimate 2010 | NRS |
| **Population Projections 2020/2030** | Used NISRA specific assumptions ( 2010 based projections) | NISRA | M2F2 assumption | PHIS (health well)- CSO | Used NRS specific assumptions | NRS |

**Table B: Data sources used in projecting CHD mortality - definitions and data sources for NI, RoI & Scotland**

*physical inactivity data based on IPAQ questionnaire ^5^ **NANS** - National Adult Nutrition Survey,  **TILDA** The Irish Longitudinal Study on Ageing **HSfE** - Health Survey for England **HSNI** - Health Survey Northern Ireland **FFS** - Family food survey **NISRA** – Northern Ireland Statistics & Research Agency **SLAN** – Survey of Lifestyle, Attitudes & Nutrition in Ireland **CSO** - Central Statistics Office **SHeS** - Scottish Health Survey **NRS** – National Records of Scotland

The country specific data sources listed in Table B can be accessed as outlined below:

**S4A. Northern Ireland**

Population estimates, population projections and CHD mortality are routinely published and can be accessed at: <http://www.nisra.gov.uk/demography/default.asp.htm>;

Both risk factor data on smoking and physical activity sourced from the 2010/11 Northern Ireland Health survey and hypertension data sourced from the 2010 Health Survey for England can be accessed by researchers via the UK data archive: <http://www.data-archive.ac.uk/>;

Mean Salt consumption was sourced from the family food survey which can be accessed at:

<http://webarchive.nationalarchives.gov.uk/20130103014432/http://www.defra.gov.uk/statistics/foodfarm/food/familyfood>;

Systolic blood pressure data was accessed from the National Adult Nutrition Survey <http://www.ucc.ie/en/nans/> and the Irish Longitudinal Study on Ageing which can be accessed by researchers at <https://www.ucd.ie/issda/data/>

Cholesterol data was accessed from the Survey of Lifestyle, Attitudes & Nutrition in Ireland (SLAN) which can be accessed by researchers at which can be accessed at <https://www.ucd.ie/issda/data/>

**S4B. Republic of Ireland**

Population estimates, population projections and CHD mortality are routinely published and can be accessed at: <http://www.cso.ie/en/index.html>

Risk factor data on smoking, physical activity, mean salt intake and cholesterol were sourced from the Survey of Lifestyle, Attitudes & Nutrition in Ireland (SLAN) accessible to researchers via <https://www.ucd.ie/issda/data/>

Blood pressure data was sourced from the National Adult Nutrition Survey which can be accessed at <http://www.ucc.ie/en/nans/>

Blood pressure and smoking data obtained from The Irish Longitudinal Study on Ageing can be acceesed by researchers via <https://www.ucd.ie/issda/data/>

**S4C. Scotland**

Population estimates, population projections and CHD mortality are routinely published and can be accessed at <http://nationalrecordsofscotland.gov.uk/>:

Risk factor data on smoking, physical activity and blood pressure and cholesterol were sourced from Scottish Health Surveys which can be accessed by researchers via the UK data archive: <http://www.data-archive.ac.uk/> &

Mean Salt consumption was sourced from the Scottish urinary 2009 survey and can be accessed at:

<http://www.food.gov.uk/sites/default/files/681-1-1229_S14047.pdf>

|  |  | **NI** | | **RoI R.Ireland** | | **Scotland** | |
| --- | --- | --- | --- | --- | --- | --- | --- |
|  |  | **Males** | **Females** | **Males** | **Females** | **Males** | **Females** |
| **Mean SBP** | **25-34** | 129.0 | 111.0 | 129.0 | 111.0 | 126.7 | 114.1 |
|  | **35-44** | 128.0 | 114.0 | 128.0 | 114.0 | 130.9 | 118.5 |
|  | **45-54** | 132.0 | 118.0 | 132.0 | 118.0 | 130.5 | 125.4 |
|  | **55-64** | 129.8 | 139.1 | 129.8 | 139.1 | 135.2 | 132.4 |
|  | **65-74** | 137.5 | 141.9 | 137.5 | 141.9 | 138.0 | 138.9 |
|  | **75-84** | 145.7 | 143.4 | 145.7 | 143.4 | 136.4 | 137.9 |
|  | **25-84** | 131.6 | 124.2 | 131.2 | 122.8 | 132.1 | 126.3 |
| **Mean Cholesterol** | **25-34** | 5.5 | 5.0 | 5.5 | 5.0 | 4.8 | 4.8 |
|  | **35-44** | 5.6 | 5.3 | 5.6 | 5.3 | 5.5 | 5.2 |
|  | **45-54** | 5.3 | 5.5 | 5.3 | 5.5 | 5.6 | 5.6 |
|  | **55-64** | 4.5 | 5.0 | 4.5 | 5.0 | 5.3 | 6.0 |
|  | **65-74** | 5.2 | 4.6 | 5.2 | 4.6 | 5.0 | 5.5 |
|  | **75-84** | 5.0 | 4.3 | 5.0 | 4.3 | 4.5 | 5.6 |
|  | **25-84** | 5.4 | 5.1 | 5.4 | 5.1 | 5.2 | 5.4 |
| **Hypertension (%)** | **25-34** | 5.8 | 3.9 | 14.2 | 2.3 | 17.9 | 3.4 |
|  | **35-44** | 24.6 | 9.8 | 19.1 | 3.1 | 27.6 | 8.6 |
|  | **45-54** | 36.7 | 25.8 | 61.4 | 34.4 | 26.8 | 21.7 |
|  | **55-64** | 50.6 | 46.5 | 70.8 | 56.4 | 34.5 | 36.4 |
|  | **65-74** | 65.4 | 63.1 | 74.8 | 67.4 | 47.8 | 44.8 |
|  | **75-84** | 79.4 | 79.4 | 82.6 | 73.6 | 44.5 | 47.9 |
|  | **25-84** | 35.9 | 30.3 | 43.1 | 28.6 | 30.6 | 24.1 |
| **Smoking**  **(%)** | **25-34** | 32.2 | 35.2 | 39.9 | 32.3 | 34.2 | 28.8 |
|  | **35-44** | 25.7 | 23.5 | 36.7 | 28.5 | 30.6 | 27.9 |
|  | **45-54** | 26.0 | 25.3 | 24.7 | 30.4 | 28.1 | 27.1 |
|  | **55-64** | 23.9 | 22.8 | 21.4 | 21.8 | 23.9 | 24.5 |
|  | **65-74** | 17.1 | 13.7 | 17.3 | 15.9 | 15.9 | 18.5 |
|  | **75-84** | 5.9 | 7.3 | 9.7 | 13.7 | 13.4 | 11.8 |
|  | **25-84** | 24.6 | 23.6 | 29.5 | 26.5 | 26.4 | 24.5 |
| **Physical Inactivity**  **(%)** | **25-34** | 27.2 | 35.1 | 33.8 | 42.6 | 39.3 | 58.2 |
|  | **35-44** | 27.5 | 36.6 | 35.7 | 41.8 | 49.0 | 57.1 |
|  | **45-54** | 38.4 | 44.9 | 34.9 | 40.7 | 56.1 | 61.8 |
|  | **55-64** | 45.8 | 49.0 | 40.6 | 43.2 | 64.4 | 69.9 |
|  | **65-74** | 51.4 | 58.1 | 42.9 | 52.1 | 78.9 | 80.9 |
|  | **75-84** | 66.3 | 78.0 | 58.3 | 67.6 | 88.7 | 90.2 |
|  | **25-84** | 38.1 | 46.2 | 27.3 | 30.7 | 58.2 | 67.2 |
| **Salt (g/day)** | **25-84** | 7.8 | | 8.0 | | 8.8 | |

**Table C: Gender Specific risk factor levels in NI, RoI and Scotland by agegroup**

**S5. Translating changes in Salt and saturated fat intake into CHD mortality reductions**

The original IMPACT model had no functionality to calculate DPPs according to changes in salt consumption and saturated/unsaturated fatty acids intake. For this project, we extended the model with two additional layers to translate the effects of changes in these risk factors to changes in blood pressure and total cholesterol levels. Translating the effect of salt consumption to changes in blood pressure was based on data published in a Cochrane systematic review^6^ which quantified the effect of salt reduction on blood pressure in hypertensive and normotensive patients. According to this publication, the change in salt consumption by 6g/d results in change in systolic blood pressure by mean value of

- 1. mmHg (95%CI 5.6-8.8 mmHg) in hypertensives and 3.6 mmHg (95%CI 1.9-5.2 mmHg) in normotensive patients.

In order to model the effect of saturated fats intake on serum cholesterol level we used the Clarke equations^7^ to translate a change in saturated fat intake into a change in total cholesterol levels with replacement with poly and monosaturated to keep caloric balance (assuming that for each 1% reduction in saturated fat is replaced by 0.5% mono and poly-unsaturates):

- 5% decrease in consumed saturated fats which are replaced by polyunsaturated fats results in decrease in total cholesterol level by 0.39 mmol/L
- 5% decrease in consumed saturated fats which are replaced by monounsaturated fats results in decrease in total cholesterol level by 0.24 mmol/L

Consistent with original IMPACT methodology, the number of CHD deaths potentially prevented to 2030, as a result of improved levels of systolic blood pressure ( or cholesterol) in the population, was then estimated as the product of three variables: the number of expected future CHD mortality deaths in 2030 , the projected absolute reduction in blood pressure (or cholesterol) between 2010 and 2030 and the adjusted regression coefficient quantifying the independent relationship between population change in blood pressure and the consequent change in mortality from CHD. Regression beta coefficients for blood pressure and cholesterol are provided in Tables D and E respectively.

## S5A: Translating changes in Smoking and physical inactivity into CHD mortality reductions

Using regression coefficients from the literature ^8,9^ a population attributable risk fraction (PARF) approach was used to determine the number of deaths prevented in 2030 resulting from alternative improved future smoking and physical activity levels. The PARF was calculated conventionally for 2010 and 2030 as:  **(P x (RR-1)) / (1+P x (RR-1))**

where P is the prevalence of the risk factor and RR is the relative risk for CHD mortality associated with the individual risk factor. The number of deaths prevented was calculated as the number of deaths in 2030 multiplied by the decrease in PARF between 2010 and 2030.

**Example A: estimation of DPPs from risk factor change using PARF method.**

**Smoking in Scottish men aged 65-74 years reduces by 5% between 2010 & 2030 (lower mortality scenario)**

If the prevalence of smoking among men aged 65-74 years was 15.9% in 2010 and 10.9% in 2030. Assuming a Relative Risk of 1.68, (Table G) the PARF was 0.099 in 2010 and 0.070 in 2030. The number of deaths prevented or postponed attributable to the decrease in smoking prevalence from 2010 to 2030 is:

the projected CHD deaths in 2030, (391) * (0.0992 - 0.0702) = 11 DPPs

This calculation was then repeated

a) for men and women in each age group,

b) for physical inactivity using separate relative risks (Table H )

c) using maximum and minimum values in each group, to generate a sensitivity analysis

| **Systolic blood pressure** |  |  | **Age group (years)** | | |
| --- | --- | --- | --- | --- | --- |
|  | **25-44** | **45-54** | **55-64** | **65-74** | **75+** |
| **Men** (log hazard ratio per 1 mmHg) | **-0.036** | **-0.035** | **-0.032** | **-0.027** | **-0.021** |
| ***Minimum*** | *-0.029* | *-0.028* | *-0.026* | *-0.022* | *-0.017* |
| ***Maximum*** | *-0.043* | *-0.042* | *-0.039* | *-0.032* | *-0.025* |
| **Women** (log hazard ratio per 1 mmHg) | **-0.046** | **-0.046** | **-0.035** | **-0.032** | **-0.026** |
| ***Minimum*** | *-0.037* | *-0.037* | *-0.028* | *-0.026* | *-0.021* |
| ***Maximum*** | *-0.055* | *-0.055* | *-0.042* | *-0.039* | *-0.031* |

**Table D:** **Beta coefficients for blood pressure change in population**

Source: Prospective studies collaborative meta-analysis, Lancet 2002 ^10^

Units: Percentage change in CHD mortality per 20 mmHg change in systolic blood pressure

**Strengths:** Large dataset, includes US data, adjusted for regression dilution bias, consistent with randomised controlled trials, results stratified by age and sex, with 95% confidence intervals

**Limitations:** Some publication bias still possible

**Cholesterol Age groups (years)**

|  | **25-44** | **45-54** | **55-64** | **65-74** | **75-84** | **85+** |
| --- | --- | --- | --- | --- | --- | --- |
| **Mortality reduction per 1 mmol/l** | | | | | | |
| **Men** | 0.55 | 0.53 | 0.36 | 0.21 | 0.21 | 0.21 |
| **Women** | 0.57 | 0.52 | 0.35 | 0.23 | 0.23 | 0.23 |
| **Log coefficient** | | | | | | |
| **Men** | **-0.799** | **-0.755** | **-0.446** | **-0.236** | **-0.117** | **-0.083** |
| *Minimum* | *-0.639* | *-0.604* | *-0.357* | *-0.189* | *-0.093* | *-0.067* |
| *Maximum* | *-0.958* | *-0.906* | *-0.536* | *-0.283* | *-0.140* | *-0.100* |

| **Women** | **-0.844** | **-0.734** | **-0.431** | **-0.261** | **-0.174** | **-0.051** |
| --- | --- | --- | --- | --- | --- | --- |
| *Minimum* | *-0.675* | *-0.587* | *-0.345* | *-0.209* | *-0.139* | *-0.041* |
| *Maximum* | *-1.013* | *-0.881* | *-0.517* | *-0.314* | *-0.209* | *-0.062* |

**Table E:** **Beta coefficients for total cholesterol change in population**

Source: Prospective studies collaborative meta-analysis, Lancet 2007 ^11^ Units: Percentage change in CHD mortality

per 1 mmol/l change in total cholesterol

**Strengths:** Includes US data, adjusted for regression dilution bias, includes randomised controlled trials, RCT values consistent with observational data, results stratified by age and sex, with 95% confidence intervals

**Limitations:** Some publication bias still possible

| **Age** | **Men** | **Women** |
| --- | --- | --- |
| **30-44** | 5.51 (2.47-12.25) | 2.26 (0.83-6.14) |
| **45-59** | 3.04 (2.66-3.48) | 3.78 (3.10-4.62) |
| **60-69** | 1.88 (1.70-2.08) | 2.53 (2.22-2.87) |
| **70-79** | 1.44 (1.27-1.63) | 1.68 (1.46-1.93) |
| **>=80 years** | 1.05 (0.78-1.43) | 1.38 (1.08-1.77) |

**Table F:** **Relative risk of mortality from Ischaemic Heart Disease for current smokers relative to non-smokers (95% CIs in parentheses), from the American Cancer Society’s Cancer Prevention Study (CPS-II)**

Notes: CPS-II is an ongoing prospective study of mortality in 1.2 million Americans aged 30 years or more when they

completed a questionnaire on tobacco and alcohol use, diet, and multiple other factors affecting health and mortality in 1982. RRs were estimated from Cox proportional-hazard models, with non-smokers as the reference group (RR=1.0 for non-smokers). Risks were adjusted for age, race, education, marital status, "blue collar" employment in most recent or current job, weekly consumption of vegetables and citrus fruit, vitamin (A, C, and E) use, alcohol use, aspirin use, body mass index, exercise, dietary fat consumption and for hypertension and diabetes (both at baseline). Analyses of the hazards associated with smoking were based on the first six years of follow-up (1982 through 1988). Source: Ezzati et al (2005) ^8^

| **Age** | **Inactive level** | **Insufficiently active level** |
| --- | --- | --- |
| **15-69** | 1.71 (1.58-1.85) | 1.44 (1.28-1.62) |
| **70-79** | 1.50 (1.38-1.61) | 1.31 (1.17-1.48) |
| **80+ years** | 1.30 (1.21-1.41) | 1.20 (1.07-1.35) |

**Table G:** **Relative risk of Ischaemic Heart Disease from physical (in)activity levels from WHO GBD Study (95% CIs in parentheses), relative to those considered physically active**

Notes: Physical (in)activity in the WHO GBD study was treated as a categorical variable with three categories: Level

1: Inactive: doing no or very little physical activity at work, at home, for transport, or during discretionary time. Level 2: Insufficiently active: doing some physical activity but less than 150 minutes of moderate-intensity physical activity or 60 minutes of vigorous-intensity physical activity a week accumulated across work, home, transport or discretionary domains. Level 3: Sufficiently active (unexposed): at least 150 minutes of moderate-intensity physical activity or 60 minutes of vigorous-intensity physical activity a week accumulated across work, home, transport or discretionary domains, which approximately corresponds to current recommendations in many countries. RR estimates were adjusted for confounding variables, measurement error associated with self-report, and attenuated over age (25% of the excess risk for the 70-79 year age-group and 50% of the excess risk for the oldest age group, 80+), but not adjusted for blood pressure and cholesterol.

Sources: Bull et al (2004) [[7];](#_bookmark77) Joubert et al (2007) **^9^**

|  | **Predicted percentage* DPP**  **Assumed No Mortality Change, 2030** | | |
| --- | --- | --- | --- |
|  | **NI** | **RoI** | **Sco** |
| **Ideal Scenarios** |  |  |  |
| **Smoking [-15%]** | 6.6 (2.0-11.6) | 7.0 (2.0-12.3) | 7.5 (2.2-12.9) |
| **Salt [-30%]** | 5.4 (3.4-8.0) | 5.7 (3.7-8.0) | 5.3 (3.4-7.5) |
| **Sat/ Unsat fats [-6%]** | 8.8 (6.3-11.7) | 8.4 (6.2-11.0) | 8.8 (6.5-11.5) |
| **Phy Inactivity [-15%]** | 3.6 (0.5-6.6) | 3.8 (0.5-6.5) | 3.2 (0.5-4.6) |
| **Modest Scenarios** |  |  |  |
| **Smoking [-5%]** | 2.7 (0.8-4.7) | 2.6 (0.7-4.4) | 2.6 (0.8-4.4) |
| **Salt [-10%]** | 2.0 (1.2-2.9) | 2.1 (1.3-3.0) | 1.9 (1.2-2.8) |
| **Sat/ Unsat fats [-2%]** | 3.4 (2.4-4.5) | 3.2 (2.4-4.2) | 3.4 (2.5-4.4) |
| **Phy Inactivity [-5%]** | 1.3 (-0.4-3.2) | 1.3 (-0.1-2.9) | 1.1 (0.2-1.8) |
| **Expected Deaths** | **646 (439-860)** | **1856 (705-3085)** | **2562 (1324-3916)** |

**Table H: Predicted decreases in deaths expressed as a percentage of total expected CHD deaths in 2030 – Ideal & Modest Future Risk factor Scenarios**

|  |  | **Predicted percentage* DPP**  **Assumed lower mortality in 2030** | | |
| --- | --- | --- | --- | --- |
|  |  | **NI** | **RoI** | **Sco** |
| **Ideal Scenarios** |  |  |  |  |
| **Smoking [-15%]** | Men  women | 5.4 (1.4-10.0) | 5.4 (-0.9-13.7) | 6.7 (1.4-13.2) |
|  | Women | 6.9 (1.8-13.4) | 8.0 (-1.0-18.6) | 8.0 (1.8-16.3) |
| **Salt [-30%]** | Men  Women | 5.1 (3.0-7.7) | 5.4 (1.5-10.2) | 4.9 (2.3-8.3) |
|  | Women | 5.9 (3.4-9.1) | 5.9 (1.8-10.8) | 5.7 (2.5-9.9) |
| **Sat/ Unsat fats [-6%]** | Men  women | 8.5 (5.3-12.3) | 7.7 (1.2-15) | 9.0 (4.6-14.3) |
|  | Women | 8.9 (5.1-13.2) | 8.1 (1.9-14.9) | 8.9 (3.9-14.9) |
| **Phy Inactivity [-15%]** | Men  women | 3.6 (0.4-6.3) | 3.7 (-0.4-7.9) | 3.2 (0.4-5.5) |
|  | Women | 3.4 (0.3-5.7) | 3.4 (-0.5-6.9) | 3.0 (0.3-5.2) |
| **Modest Scenarios** |  |  |  |  |
| **Smoking [-5%]** | Men  women | 2.3 (0.5-4.2) | 2.1 (-0.3-5.1) | 2.3 (0.5-4.5) |
|  | Women | 3.1 (0.9-5.8) | 2.8 (-0.2-6.4) | 3.0 (0.7-6.0) |
| **Salt [-10%]** | Men  women | 1.8 (1.1-2.8) | 2.0 (0.5-3.7) | 1.8 (0.8-3.1) |
|  | Women | 2.2 (1.2-3.4) | 2.2 (0.6-4.0) | 2.1 (0.9-3.7) |
| **Sat/ Unsat fats [-2%]** | Men  women | 3.3 (2-.04.8) | 2.9 (0.4-5.7) | 3.5 (1.8-5.6) |
|  | Women | 3.4 (1.9-5.1) | 3.0 (0.7-5.7) | 3.4 (1.5-5.7) |
| **Phy Inactivity [-5%]** | Men  women | 1.3 (0.1-2.1) | 1.3 (-0.1-2.7) | 1.1 (0.1-1.9) |
|  | Women | 1.2 (0.1-1.9) | 1.2 (-0.2-2.4) | 1.0 (0.1-1.8) |
| **Expected Deaths** | **Men** | **461 (408-509)** | **1248 (887-1627)** | **1694 (1428-1772)** |
|  | **Women** | **185 (166-200)** | **525 (412-685)** | **868 (668-950)** |

**Table I: Predicted decreases in deaths by gender expressed as a percentage of total expected CHD deaths in 2030 – Ideal & Modest Future Risk factor Scenarios (Assumed Lower mortality)**

|  |  | **Predicted percentage* DPP**  **Assumed no mortality change in 2030** | | |
| --- | --- | --- | --- | --- |
|  |  | **NI** | **RoI** | **Sco** |
| **Ideal Scenarios** |  |  |  |  |
| **Smoking [-15%]** | Men  women | 6.1 (1.8-10.6) | 6.2 (1.7-11.1) | 6.9 (1.9-12.0) |
|  | Women | 7.6 (2.3-13.7) | 9.0 (2.7-15.4) | 8.5 (2.8-14.4) |
| **Salt [-30%]** | Men  Women | 5.2 (3.2-7.5) | 5.5 (3.7-7.8) | 5.0 (3.2-7.2) |
|  | Women | 6.0 (3.7-8.8) | 6.0 (3.9-8.5) | 5.7 (3.7-8.3) |
| **Sat/ Unsat fats [-6%]** | Men  women | 8.8 (6.3-11.7) | 8.4 (6.2-10.9) | 8.9 (6.6-11.5) |
|  | Women | 8.9 (6.2-11.9) | 8.5 (6.3-11.0) | 8.8 (6.5-11.5) |
| **Phy Inactivity [-15%]** | Men  women | 3.8 (0.5-6.1) | 3.9 (0.4-6.4) | 3.2 (0.5-4.7) |
|  | Women | 3.4 (0.5-5.4) | 3.5 (0.5-5.5) | 3.0 (0.5-4.3) |
| **Modest Scenarios** |  |  |  |  |
| **Smoking [-5%]** | Men  women | 2.4 (0.6-4.2) | 2.3 (0.6-4.1) | 2.4 (0.6-4.0) |
|  | Women | 3.3 (1.0-5.7) | 3.1 (1.0-5.2) | 3.1 (1.1-5.2) |
| **Salt [-10%]** | Men  women | 1.9 (1.2-2.8) | 2.0 (1.3-2.9) | 1.8 (1.2-2.6) |
|  | Women | 2.2 (1.3-3.2) | 2.2 (1.4-3.2) | 2.1 (1.3-3.1) |
| **Sat/ Unsat fats [-2%]** | Men  women | 3.4 (2.4-4.5) | 3.2 (2.4-4.2) | 3.4 (2.5-4.4) |
|  | Women | 3.3 (2.3-4.5) | 3.2 (2.3-4.2) | 3.3 (2.4-4.4) |
| **Phy Inactivity [-5%]** | Men  women | 1.3 (0.2-2.1) | 1.3 (0.2-2.2) | 1.1 (0.2-1.6) |
|  | Women | 1.2 (0.2-1.9) | 1.2 (0.2-1.9) | 1.1 (0.2-1.5) |
| **Expected Deaths** | **Men** | **1488 (1303-1635)** | **3829 (3429-4426)** | **5042 (4933-5497)** |
|  | **Women** | **718 (526-754)** | **1601 (1467-1715)** | **2736 (2362-3065)** |

**Table J: Predicted decreases in deaths by gender expressed as a percentage of total expected CHD deaths in 2030 – Ideal & Modest Future Risk factor Scenarios (Assumed no mortality change)**

**Figure B: Predicted decreases in Deaths in 2030 based on a ‘No mortality change’ between 2010 and 2030 for (A) IDEAL SCENARIOS & (B) MODEST Scenarios ( the error bars shows the extreme minimum and maximum values in the sensitivity analysis)**

Projected DPP’s for England and wales have been estimated based on simple extrapolation of both the Northern Irish (extrapolation 1) and Scottish (extrapolation 2) DPP’s.

Table K: Extrapolation of modelled estimates to the British Isles population for 2030

***Based on NI DPP’s, **Based on Scotland DPP’s**

## S6. Sensitivity analysis parameters

| Group | Parameters | Distribution | Distribution parameters |
| --- | --- | --- | --- |
| Population counts in base year and CHD deaths stratified by age and sex | Population counts (no error) | No error (uniform distribution) |  |
|  | CHD mortality (no error) | No error (uniform distribution) |  |
| Population counts in final year stratified by age and sex | Population counts | Normal(mean, SD) | Mean =point estimate SD=standard error of the mean |
|  | CHD mortality | Normal(mean, SD) | Mean =point estimate SD=standard error of the mean |
| Prevalence/mean estimates | Prevalence estimates (smoking physical activity, hypertension prevalence) – beta distribution. | Beta (alpha, beta) | Alpha=casesBeta= non-cases |
|  | Continuous variables (SBP, total cholesterol, salt intake) | Normal(mean, SD) | Mean =point estimateSD=standard error of the mean |
| Relative risk reduction | Relative risk for CHD deaths forsmoking and physical incativity | RelRisk(RR, SE ln(RR)) | RR=relative riskSE ln(RR) =standard error |
| Beta coefficients | Beata coefficients for quantifying relation of SBP and cholesterol level with CHD mortality | Normal (mean, SD) | Mean =point estimateSD=standard error of the mean |

Table L: Distributions used for main input parameters in the model.

**S7. REFERENCES**

1. Hughes J, Kee F, O'Flaherty M, Critchley J, Cupples M, Capewell S, et al. Modelling coronary heart disease mortality in Northern Ireland between 1987 and 2007: broader lessons for prevention*. Eur J Prev Cardiol* 2013; Apr;20(2):310-21.

2. Kabir Z, Perry IJ, Critchley J, O'Flaherty M, Capewell S, Bennett K. Modelling Coronary Heart Disease Mortality declines in the Republic of Ireland, 1985-2006*. Int J Cardiol* 2013; Mar 27;.

3. [Kabir Z](http://www.ncbi.nlm.nih.gov/pubmed?term=Kabir%20Z%5BAuthor%5D&cauthor=true&cauthor_uid=23541608), [Perry IJ](http://www.ncbi.nlm.nih.gov/pubmed?term=Perry%20IJ%5BAuthor%5D&cauthor=true&cauthor_uid=23541608), [Critchley J](http://www.ncbi.nlm.nih.gov/pubmed?term=Critchley%20J%5BAuthor%5D&cauthor=true&cauthor_uid=23541608), [O'Flaherty M](http://www.ncbi.nlm.nih.gov/pubmed?term=O'Flaherty%20M%5BAuthor%5D&cauthor=true&cauthor_uid=23541608), [Capewell S](http://www.ncbi.nlm.nih.gov/pubmed?term=Capewell%20S%5BAuthor%5D&cauthor=true&cauthor_uid=23541608), [Bennett K](http://www.ncbi.nlm.nih.gov/pubmed?term=Bennett%20K%5BAuthor%5D&cauthor=true&cauthor_uid=23541608). Modelling Coronary Heart Disease Mortality declines in the Republic of Ireland, 1985-2006. [Int J Cardiol.](http://www.ncbi.nlm.nih.gov/pubmed/23541608) 2013 Oct 3;168(3):2462-7. doi: 10.1016/j.ijcard.2013.03.007. Epub 2013 Mar 28.

4. Capewell S, Morrison CE, McMurray JJ. Contribution of modern cardiovascular treatment and risk factor changes to the decline in coronary heart disease mortality in Scotland between 1975 and 1994*. Heart* 1999; Apr;81(4):380-6.

5. <http://www.ipaq.ki.se/scoring.pdf> International Physical Activity Questionnaire, 2013.

6. He, F J, The Effect of longer-term modest salt reduction on blood pressure, The Cochrane database of systematic reviews, 2004(3):CD004937

7. Clarke R, Frost C, Collins R, Appleby P, Peto R. Dietary lipids and blood cholesterol: quantitative meta-analysis of metabolic ward studies*. BMJ*1997; Jan 11;314(7074):112-7.

8. Ezzati M, Henley SJ, Thun MJ, Lopez AD. Role of smoking in global and regional cardiovascular mortality. Circulation. 2005 Jul 26;112(4):489-97.

9. Bull F, Armstrong TP, Dixon T, Ham S, Neiman A, et al. (2004) Physical inactivity. In: Ezatti M, Lopez AD, Rodgers A, Murray CJL, editors. Comparative quantification of risk. Global and regional burden of disease at- tributable to selected major risk factors. Volume 1 ed. Geneva: World Health Organization. pp. 729-8818.

10. Lewington S, Clarke R, Qizilbash N, Peto R, Collins R, Age-specific relevance of usual blood pressure to vascular mortality: a meta-analysis of individual data for one million adults in 61 prospective studies. Lancet. 2002 Dec 14;360(9349):1903-13.

11. Lewington S, Whitlock G, Clarke R, Sherliker P, Emberson J, Halsey J, Qizilbash N, Peto R, Collins R. Blood cholesterol and vascular mortality by age, sex, and blood pressure: a meta-analysis of individual data from 61 prospective studies with 55,000 vascular deaths. Lancet. 2008 Jul 26;372(9635):292.
